# A semantic FAIRness framework for epidemiological analysis of COVID-19 data in the UAE

**DOI:** 10.3389/fpubh.2026.1759032

**Published:** 2026-06-02

**Authors:** Haleema Al Sabbah, Anoud Bani Hani, Nawel Bessadet, Olatunde Aremu

**Affiliations:** 1 Abu Dhabi University, Abu Dhabi, United Arab Emirates; 2 University of Colorado Denver, Denver, CO, United States; 3 Department of Public Health, College of Health Sciences, Birmingham City University, Birmingham, United Kingdom

**Keywords:** automated data linkage, COVID-19, data analysis, epidemiological analysis, FAIR data, semantic knowledge graphs

## Abstract

The increasing availability of Coronavirus disease 2019 (COVID-19)–related data has highlighted the need for robust epidemiological analysis to support public health decision-making, particularly in contexts where data are heterogeneous and fragmented. In the United Arab Emirates (UAE), COVID-19 research has generated diverse genomic, clinical, and epidemiological datasets, yet their integration and reuse remain challenging due to inconsistencies in data representation, semantics, and interoperability. This study aimed to review key genomic and epidemiological studies related to COVID-19 in the UAE and, informed by identified gaps, proposes a semantic FAIRness framework for epidemiological data integration and analysis. The framework leverages the FAIR data principles and semantic technologies to provide a conceptual architecture for aggregating heterogeneous data sources, transforming data using ontological models, and enabling semantic linkage and reasoning across datasets. At a conceptual level, the framework is intended to support comparative analysis across studies, facilitate transparent representation of uncertainty, and promote semantically interoperable data sharing among diverse stakeholders. While selected components of the framework build on prior proof-of-concept implementations, the framework as a whole has not yet been fully implemented or empirically evaluated. The proposed approach is therefore positioned as a foundation for future development and evaluation, with the potential to enhance evidence-informed epidemiological analysis and public health decision-making in the UAE and similar contexts.

## 1 Introduction

The Coronavirus disease 2019 (COVID-19) pandemic overwhelmed health systems throughout the world and has led to the death of millions of individuals. It persists, and SARS-CoV-2, the virus that causes it, changes over time (WHO, 2022). According to the World Health Organization (WHO) (2022), most changes have little to no impact on properties of the virus, but some changes may affect features such as ease of spread, disease severity, vaccine performance, therapeutic medicines, diagnostic tools, or other public health and social measures. Hence, there have been efforts to monitor and assess the evolution of SARS-CoV-2 since it emerged. Researchers and medical experts continue to use different approaches to analyze the virus to understand the epidemiological behavior of its genetic variants. Researchers seek to understand the COVID-19 genome, its behavior, and its functions to develop an effective vaccine or drug. Genome sequence analysis guides clinical experts to provide further personalized therapeutic or diagnostic decision- making strategies. When researchers unlock the genetic information of a virus and its impacts, it is hoped to inform public health, enhance decision-making, and find effective treatments. Genome analysis of coronavirus and its variants informs their behavior, characteristics, development history, detection, and fosters the development of treatment and vaccines (1). Genomic analysis also aids in detecting its mutations and their possible effect on its spread and potential predictions (2, 3). There have been efforts to bring together sequence data of population-level and genomics data with phenotypic knowledge, clinical reports, and other types of multi-omics datasets globally, including within the UAE. This is investigative research that produces complex scientific and computational demands. Hence, it is necessary to create computational techniques capable of enhancing the evaluation of extensive high- dimensional and heterogeneous datasets. The adoption of semantic technologies also foster identification of actionable insights and enhance the overall response to the public health concerns caused by COVID-19 in UAE. Adopting semantic technology can enhance the achievement of the four foundational principles of generating and publishing data, including Findability, Accessibility, Interoperability, and Reusability (FAIR), thereby maximizing the data, as well as workflows, tools, and algorithms that produce such data (4). This study aims to use the semantic FAIRness framework to enhance epidemiological analysis of COVID-19 data in the UAE. The framework facilitates this by leveraging semantic techniques for the aggregation of data from multiple heterogeneous sources, transformation of the data based on a unifying ontological model, defining automated links and relationships between the data, fostering data reasoning and insights generation, thereby ensuring the achievement of data FAIRness principles to increase data quality for decision-making by diverse types of stakeholders, including government agencies, public health professionals, data scientists, and others. For the remainder of this study, Section 2 investigates and reviews existing research in the domain with a view to identifying current gaps in research. Section 3 focuses on the main findings from the literature review and provides a critical analysis of these. Section 4 describes the design and development of a proposed semantic FAIR framework for epidemiological analysis of COVID-19 data, while Section 5 focuses on further analysis of the framework. This study concludes with a summary in Section 6.

## 2 Literature review

The literature review was conducted using a targeted and systematic search strategy to identify peer-reviewed studies and preprints addressing COVID-19 genomic sequencing, phylogenetic analysis, and epidemiological investigations in the United Arab Emirates. Primary bibliographic databases searched included PubMed, Scopus, and Google Scholar. These were supplemented by specialized genomic and molecular biology repositories, notably the GISAID EpiCoV database and the National Center for Biotechnology Information (NCBI), to capture studies involving viral genome sequencing and variant analysis.

The search covered publications from the onset of the COVID-19 pandemic through its peak period, thereby encompassing the initial emergence of severe acute respiratory syndrome coronavirus 2 (SARS-CoV-2) and the subsequent variants of concern. Key search terms and combinations included “COVID-19,” “SARS-CoV-2,” “United Arab Emirates,” “UAE,” “genomic epidemiology,” “phylogenetic analysis,” “semantic FAIR data,” and “clinical outcomes.” Studies were selected based on their relevance to genomic, epidemiological, or clinical analyses conducted in or involving the UAE. This approach enabled the inclusion of diverse study designs, ranging from trans-ancestry genome-wide association studies to descriptive clinical and epidemiological characterizations. It provided a comprehensive foundation for identifying research gaps and informing the proposed semantic FAIRness framework.

The studies reviewed in this manuscript are most closely aligned with applied public health epidemiology practices as articulated by organizations such as the WHO and CDC, and with community-governed standards for viral genome sequencing and data sharing established through platforms such as GISAID and NCBI. Observational and genetic analyses within this body of work also reflect broader biomedical research conventions, but the dominant methodological orientation is toward public health surveillance and genomic epidemiology rather than formal reporting checklists.

Public health data needs to be shared, reused, integrated, analyzed, and interpreted, most especially in real-time, to aid successful interventions in times of emergencies, such as in pandemics. Digital technologies, such as those that involve semantic processing of data, offer comprehensive, specialized repositories that are curated continuously and capture high-value reference datasets. These datasets are fine-tuned to enhance research output and support users. Resources devoted to COVID-19 continue to grow as new challenges facing the efforts to stop its spread emerge. Researchers are providing information regarding the characteristics of COVID-19 and quantifying the possible impact of trials. Semantic technologies, such as the Resource Description Framework (RDF), ontology, and knowledge graphs, can make sense of COVID-19 information, such as alignment of genome sequences, count of amino acids, nucleotide composition and their frequency, tri-nucleotide compositions, their DNA similarity information, and more (5).

This study focuses on data describing the nature of COVID-19 and its possible effect on its spread and possible predictions, as this enhances drug discoveries and the production of vaccines. It is focused on bringing such data together in the context of the UAE for FAIRness, which ensures that data is findable, accessible, interoperable, and reusable for knowledge discovery and innovation (4). Several studies reviewed in this study conducted genomic sequencing, phylogenetic analysis, and epidemiological analyses across multiple factors and variables to examine COVID-19 in the UAE.

### 2.1 Genomic sequencing and phylogenetic analysis of COVID-19 in the UAE

Authors of (6) used UAE data on COVID-19 to come up with SARS-CoV-2 full-genome sequences. Then, they analyzed them for virus introductions, chain of transmissions, and possible links to earlier strains observed elsewhere. The researchers obtained global non-UAE genome sequences from the GISAID EpiCoV database to carry out the phylogenetic analysis. Based on the phylogenetic analysis conducted in the study, SARS-CoV-2 was spatiotemporally introduced into the UAE from the Middle East, Europe, and Asia during the early phase of the pandemic. The study demonstrated early community-based transmission and the emergence of new mutations in SARS-CoV-2 strains in the UAE. Research in [7] also examined the origin of SARS-CoV-2 in the UAE, its spread, and its evolution in the country. They performed meta-transcriptome sequencing on samples to identify the distribution of international clades. The study discovered eleven (11) new genetic variants, thereby defining five subclades specific to the UAE viral population. It was revealed that business activity was associated with cross-settlement human-to-human transmission. The results indicate where to look and what to consider in further studies on host–pathogen interactions. Table 1 summarizes COVID-19 genomic sequencing and phylogenetic analysis in the UAE.

**Table 1:** COVID-19 genomic sequencing and phylogenetic analysis in the UAE.

Study	UAE samples used for genome sequence	# UAE genome for phylogenetic analysis	Compares with	Global data source for genome from other countries	# genome from others countries
[1]	49	25	Global genomes	GISAID	157
[2]	1,067	637 + 25 from [1]	Global genomes	GISAID	52
B [3]	32	32	Saudi Arabia	GISAID NCBI	142

Furthermore, research in (8) obtained mRNA genome sequences data (for the United Arab Emirates and the Kingdom of Saudi Arabia) from the GISAID database for genomic analysis. The research also focused on retrieving mRNA genome references from the NCBI database to identify the closest genomes to those observed in China. The study examined genomic diversity to determine the specificity of mutations observed in the country, and likewise analyzed the epidemiological behavior. The researchers investigated the nature and pattern of the first wave. Then, they sought potential factors that could have affected the observed behaviors, such as risk factors, diversity, observed mutations, and more. The comparison between the two countries provided insights into how the pandemic was handled and the contributions of different factors.

### 2.2 Epidemiological analysis of COVID-19 in the UAE

Authors of (9) considered the association between the neurohormone vitamin D’s insufficient blood levels and increased risk of COVID-19 severity and mortality to examine the association between vitamin D status and COVID-19 severity and mortality in the UAE. They discovered a significant association with a higher risk of severe COVID-19 infection and death for some vitamin D levels. The study provides insight into the contribution of independent risk factors to the outcome.

The study by Al- (10) examined the relationship between vitamin D metabolism and COVID-19 outcomes in the UAE, as genetic variants in vitamin D metabolism have been implicated as potential risk factors for severe COVID-19 outcomes. It studied the impact of genetic variations in humans on COVID-19 clinical presentation.

Twelve (12) single-nucleotide polymorphisms associated with the critical COVID-19 condition were observed, and the researchers reported that genetic determinants of vitamin D metabolism are significantly associated with COVID-19 severity in the country. The authors of (11) conducted a trans-ancestry genome-wide association study (GWAS) meta-analysis of COVID-19 severity using patient data from the UAE. The populations considered in the trans-ancestry meta-analysis GWAS include East Asia, South Asia, America, and Europe. The researchers conducted an all-inclusive post-GWAS analysis, including enrichment of SNP associations in tissues and cell types, expression quantitative trait loci, and differential expression analysis. The study discovered eight (8) highly plausible genetic associations with hospitalized cases of COVID-19.

The description of the demographics, clinical features, and outcomes of confirmed COVID-19 cases in the UAE was provided by (12). The descriptive analysis provided insights into case notification, infection and transmission rates, comorbidities, mortality rate, and public health measures. The authors of (13) also conducted a cross-sectional descriptive study of hospitalized symptomatic COVID-19 patients in the UAE, examining demographic, comorbidities, clinical symptoms, laboratory parameters, radiologic features, clinical complications and outcomes, and medications used in the treatment of COVID-19 patients. Table 2 summarizes the main research efforts on COVID-19 epidemiological analysis in the UAE.

**Table 2:** COVID-19 epidemiological analysis in the UAE.

Study	Study sample	Process	Method(s)
[4]	646	DNA Extracting and Genotyping	Statistical analysis; Genome-wide association study (GWAS)
[5]	522	Extracting and quantifying RNA	Statistical analysis
[6]	646	DNA Extracting and Genotyping	Statistical analysis; Genome-wide association study (GWAS); Gene annotation and expression quantitative trait loci (eQTL); Functional annotation.
[7]	19	Verification of co-morbidity status; data collection	Statistical analysis
[8]	525	Laboratory tests; data collection	Statistical analysis
[9]	111	Data collection; classification	Statistical analysis
[10]	1,249	Data collection; classification	Statistical analysis
[11]	3,452	Data collection; classification	Statistical analysis
[12]	391	Data collection; classification	Statistical analysis

Authors of (14) investigated the clinical characteristics, laboratory findings, treatment, and outcomes of children with COVID-19. The research examined the demographics, premorbid clinical characteristics, and inpatient hospital courses, concluding that most children did not experience any symptoms and that severe COVID-19 disease is not common in the UAE.

Authors of (15) analyzed self-reported sociodemographic characteristics, employment status, travel history, and chronic comorbidities of COVID-19 cases in the UAE with a focus on the epidemiological characterization of symptomatic and asymptomatic cases. Authors of (16) carried out a retrospective extraction of UAE COVID-19 patient characteristics, comorbidities, and clinical outcomes data from electronic patient medical records for analysis, thereby suggesting that people older than 51 years and those with kidney disease prompted a significant increase in mortality in COVID-19 patients. Likewise, it shows an insignificant association between ethnicity and mortality in the UAE population. The authors of (17) analyzed data on COVID-19 patients in the UAE, concluding that patients had pre-existing conditions and were young; ferritin, CRP, and D-dimer levels were higher in non-survivors.

### 2.3 Gap analysis

There is a huge amount of information related to COVID-19 across multiple platforms and public repositories aimed at facilitating sharing. Epidemiological analyses of the virus have considered the link between comorbidities like obesity, hypertension, type 2 diabetes, cardiovascular diseases, and susceptibility to the development of severe COVID-19 symptoms. Numerous studies have been able to associate COVID-19 severity and fatality with several host factors, including age, gender, race, ethnicity, and presence of other comorbidities (18).

In the United Arab Emirates, research efforts focus on understanding the COVID-19 profile to contain the disease as well as establish proper guidelines and strategies to deal with any subsequent waves. Researchers have obtained patient data and conducted genomic sequencing, phylogenetic analysis, and epidemiological analysis of COVID-19 to understand the virus, its transmission, infection dynamics, factors surrounding interactions, and the effectiveness of management strategies in place. The identification of genetic variants related to COVID-19 susceptibility and severity could yield new biological insights into disease pathogenesis and inform mechanistic targets for therapy and vaccination. Since such efforts provide valuable information to researchers and public health officials for national outbreak responses, there is a need to establish a mechanism to ensure that they are available and accessible in real-time. The adoption of semantic technologies can provide a standardized means of producing findable, accessible, interoperable, and reusable information on the virus, thereby eliminating underestimation of the genetic diversity of viral strains and their associations with different factors. This will improve understanding of SARS-CoV-2 transmission and infection dynamics in the UAE and in relation to other countries.

Epidemiological analysis, known as descriptive epidemiology, is conducted to identify patterns among cases and populations across persons, places, and time. The patterns obtained from the analysis are used by epidemiologists to develop hypotheses about the causes of these patterns and the factors that increase the risk of disease. Epidemiologists primarily use a comparison group for epidemiological analysis to generate hypotheses. They look for the characteristic associated with a disease, and this is confirmed when there is a likelihood that persons with a certain characteristic will contract a disease relative to those without the characteristic. The characteristic could be a demographic factor (sex, race, or age); a constitutional factor (immune status or blood group); behavior or act; or circumstance (19). In sum, analytic epidemiology focuses on identifying the causes and effects of disease, as well as how and why it happens. Analytic epidemiology is used to quantify associations between outcomes and exposures and to test hypotheses regarding causal relationships. This provides sufficient evidence for public health professionals and organizations to take appropriate prevention and control measures (19).

## 3 Proposed semantic FAIRness framework

**Figure 1:**
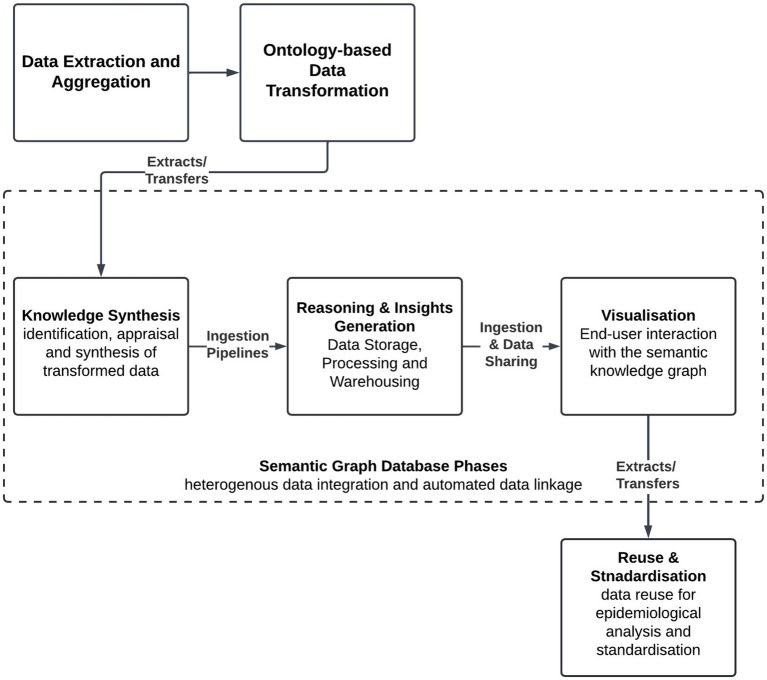
High-level processes with the semantic FAIR conceptual framework.

The proposed semantic framework leverages the principles of FAIR data and comprises of six major processes which include data extraction and aggregation, data transformation, knowledge synthesis, reasoning and generation of insights, visualization, reuse and standardization (Figure 1). These are in line with the COVID-19 ontology for cases and patient information (CODO) (20) because it was built to implement FAIR data principles. The data aggregation phase involves accessing and extracting data from heterogeneous sources and aggregating them into a common representation format for consistency and coherence in the knowledge representation. When data are aggregated, it improves the completeness of the extraction, enabling correctness of data by removing redundancies and ambiguities, thereby enhancing the trustworthiness of facts within the data (21). Data transformation involves analysis of the aggregated data to define concepts and break them into entities. The definitions and characteristics of each concept are used to analyze the data and classify them based on their similarities. The data transformation also employs ontological models to interpret the data. Figure 1 illustrates the different phases and high-level processes within the semantic framework.

**Figure 2:**
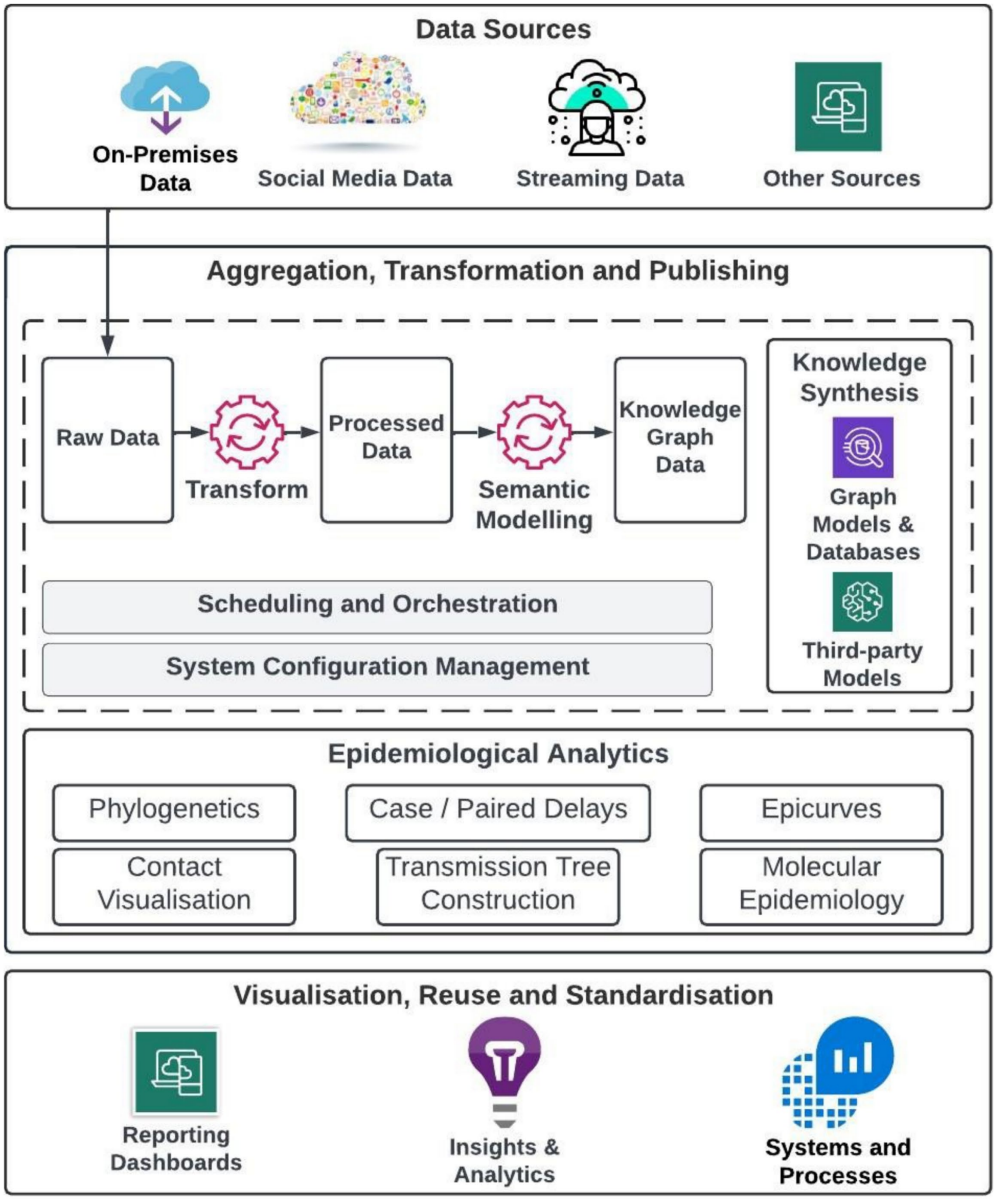
The proposed semantic FAIRness conceptual framework for epidemiological analysis of COVID-19 data.

The framework adapts YAMO (Yet Another Methodology for Ontology Development) and CODO (COVID-19 Ontology for cases and patient information) as a significant component within its semantic constituents. While CODO provides a foundation for ontological modelling in the research domain as well as leveraging FAIR data principles, YAMO defines the overall methodology to design an ontological model for COVID- 19 data. YAMO comprises ten steps, including domain identification, domain footprint, knowledge acquisition, knowledge formulation, knowledge production, term standardization, knowledge ordering, knowledge modelling, knowledge formalization, and evaluation (20). CODO, on the other hand, comprises nine development processes: the definition of purpose, derivation of competency questions, term extraction, analysis, knowledge synthesis, reuse and standardization, design of representational model, ontology development, and evaluation. The knowledge synthesis process synthesizes and organizes knowledge by defining relationships between concepts modelled in the knowledge data repository. This also enables the discovery of concept hierarchies. Reasoning and the generation of insights involve applying semantic rules to draw inferences and deduce facts from declarative knowledge from the knowledge synthesis phase. Endpoints for a wide range of end-users are developed to query the resulting knowledge graph and to provide reporting dashboards for timely knowledge acquisition and discovery. Reuse and standardization foster integration with other systems as well as re-use, by integrating concepts from various vocabularies such as Schema.org, Friend of a Friend (FOAF), SNOMED, CT, and OBO. Figure 2 presents the overall proposed semantic FAIR framework for epidemiological analysis of COVID-19 data.

The data extraction and aggregation, as well as the ontology-based data transformation processes, leverage the concept of Identity Resolution. The next three processes comprise the main semantic FAIR features of the framework, including the design and development of knowledge graphs and their storage in semantic graph databases such as Neo4j and GraphDB. The identity resolution concept defines a framework based on domain-specific, predefined matching criteria, expressed in a human-friendly way using predicate logic. This is used to efficiently extract and aggregate data from multiple sources without duplication. It also includes capabilities for extracting information and resolving semantic ambiguities across different ontologies. Furthermore, it facilitates data consolidation by identifying references to the same objects existing in different data sources and pairing them. The IdRF (Identity Resolution Framework) operates on an ontology that represents the domain, as well as known entities and properties. It resolves their identity and populates the ontology with entities with complete semantic descriptions. It continues to update the ontology to refine the identity criteria, refine the evidence calculation, and introduces new entities, thereby enhancing the identity resolution. The fundamental function of the IdRF framework is to receive an instance to update the ontology either through the insertion of new instances with their properties or updating existing ones (21). The four components of the IdRF include pre-filtering, evidence collection, decision-making, and data integration, facilitated by the implementation layer, the Semantic Description Compatibility Engine (SDCE), which provides access to the ontology. IdRF receives data from a source, and the pre-filtering component removes irrelevant parts of the ontology to produce a smaller set of instances, like the source entity. This means it preselects ontology objects eligible for identification. The evidence collection component collects evidence on the similarity between the source entity and each target in the ontology. The decision-maker determines the best identity match based on the collected evidence. The data integration component registers the incoming entity to the ontology (21). The semantic graph database handles knowledge synthesis, reasoning, and generation of insights, as well as visualization based on the connected ontology. Semantic graph databases are highly efficient and robust with RDF and SPARQL compatibility, providing triple stores for semantic inferencing at scale and facilitating the derivation of new meaning from existing data. It is capable of handling massive loads, queries, and inference in real time. It facilitates data reasoning and the generation of insights through the Reasoner known as Triple Reasoning and Rule Entailment Engine (TRREE), which stores explicit and inferred statements in highly optimized data structures, ready for query evaluation and further inference. The TRREE reasons data using a total materialization strategy based on forward-chaining of entailment rules over RDF triple patterns with variables (22).

Furthermore, the framework includes a scheduling and orchestration layer. Scheduling facilitates the initiation of tasks within the architectural design, ensuring the provisioning and allocation of computing resources as required. This, in turn, ensures efficient use of computing resources, including load balancing across multiple task instances during high-volume data processing. These are all managed and controlled via an orchestration layer. This also abstracts the entire application layer away from the underlying platform’s complexity, ensuring that tasks are deployed, managed, scaled, networked, and available in an automated manner. These all provide effective support for enhanced epidemiological analysis, including phylogenetics, which studies the interrelationships among groups of organisms in relation to evolution. It also includes support for visualizations, including epi curves for displaying the onset of illness among cases related to an outbreak; contact visualization for information relating to disease surveillance, prevention, and control, towards tracking the spread of infectious diseases within interconnected social networks. Furthermore, it enhances molecular epidemiology, which involves the integration of innovative laboratory methods into epidemiology for recognizing disease cause and facilitating intervention. It considers interactions among genetic, environmental, and other factors towards identifying susceptible populations and individuals. Likewise, supporting transmission tree construction, which involves diagrammatic representation of the chain of infections for all infected hosts and may also include information about the time each individual was infected or became infectious and when they cease to be infectious.

## 4 Operational epidemiological use cases and decision-making support

While the proposed semantic FAIRness framework is designed as a general-purpose infrastructure for epidemiological data integration and analysis, its value is best illustrated through concrete epidemiological use cases. By semantically integrating genomic, clinical, and demographic data from heterogeneous sources, the framework enables analyses that are difficult to perform reliably under current fragmented data practices. One key application is phylogenetic and molecular epidemiology analysis, where viral genome sequences can be linked with patient-level clinical outcomes and temporal metadata. This enables more precise tracking of variant introductions, community transmission patterns, and associations between viral mutations and disease severity. When combined with demographic attributes such as age, sex, occupation, or comorbidities, such analyses can support the identification of population subgroups at elevated risk from specific variants.

The framework also supports transmission tree construction and contact visualization by linking case notification data, exposure histories, and genomic similarity measures within a unified semantic model. This enables epidemiologists to reconstruct transmission chains more efficiently and to distinguish between imported cases and sustained community transmission. Such insights can inform targeted non-pharmaceutical interventions, including focused testing, isolation strategies, or workplace-specific mitigation measures. Furthermore, the integration of longitudinal case data enables the automated generation of epi curves stratified by demographic or clinical characteristics, supporting real-time monitoring of outbreak dynamics across subpopulations. For example, stratified epi curves can reveal whether infection waves disproportionately affect older population, specific occupational groups, or patients with underlying health conditions, thereby guiding targeted protection strategies.

Analogous to studies in other public health settings that have used curated multi-source datasets to quantify gaps in vaccine coverage or population immunity, the proposed framework enables similar stratified analyses in the UAE context. By linking genomic surveillance data with vaccination status, clinical severity, and comorbidity profiles, public health authorities could identify groups at risk of reduced protection or increased disease burden and adapt intervention strategies accordingly.

The framework shifts epidemiological analysis from isolated, dataset-specific investigations toward a semantically interoperable, evidence-driven workflow. This enhances analytical reproducibility, reduces uncertainty arising from data fragmentation, and supports more informed, timely, and targeted public health decision-making in response to evolving infectious disease threats (Table 3).

**Table 3:** How the framework operationally improves the analytic process.

Analytic area	Current practice (siloed)	Framework-enabled practice (semantic)
Data integration	Manual aggregation of heterogeneous source, high risk of semantic ambiguity	Automated data linkage using unifying ontological model (CODO) and identify Resolution
Phylogenetics	Fragmented genome sequences often analyzed in isolation from clinical metadata	Automated linkage of genomic similarity with patient clinical outcomes and temporal metadata
Transmission tracking	Difficulty in distinguishing between imported cases and community spread due to data gaps	Real-time transmission tree construction and contact visualization via semantic reasoning
Risk assessment	Static reporting of demographics like age or comorbidities	Dynamic, stratified epi curves that identify susceptibility populations by integrating genetic and environmental factors

The diversity of study types, reviewed in this manuscript, ranging from viral genomic sequencing and phylogenetic analyses to genome-wide association studies and clinical epidemiological investigations, maps naturally onto different layers of the proposed semantic FAIRness framework. Genomic sequencing and phylogenetic studies primarily populate the data ingestion and semantic modelling layers, where viral genomes, lineage assignments, and associated metadata are harmonized using shared ontological representations. Genome-wide association studies contribute structured genotype–phenotype associations and population-level genetic features that are integrated at the semantic integration layer, enabling linkage with clinical and demographic data. Clinical and epidemiological studies, including descriptive surveillance and outcome analyses, are represented across the integration and analytics layers, where patient-level observations, temporal trends, and stratified epidemiological indicators are combined and analyzed. The framework is intentionally designed to remain generic at the architectural level, while supporting these heterogeneous study categories through domain-specific semantic models and interoperable data representations.

## 5 Implementation status and proof-of-concept realization

The proposed semantic FAIRness framework is intended as both a conceptual reference architecture and a foundation for practical implementation. Several core components of the framework have already been implemented and evaluated as a proof of concept in COVID-19 data analysis for the UAE. In particular, prior work by the authors (8) has demonstrated the feasibility of aggregating and fusing heterogeneous epidemiological and genomic datasets using semantic technologies, including ontology-driven data transformation and identity resolution across multiple sources.

At the implementation level, selected components, such as data extraction and aggregation, ontology-based data transformation, semantic linkage, and knowledge graph construction, have been instantiated using real COVID-19 datasets and deployed on semantic graph database platforms. These implementations validate the framework’s ability to support interoperable data integration and reasoning across heterogeneous sources.

Other components of the framework, including advanced reasoning workflows, large-scale real-time orchestration, and fully integrated decision-support dashboards, are currently conceptual and represent planned extensions. These elements are designed to build upon the validated core infrastructure and are intended for future development and evaluation in collaboration with public health stakeholders. Distinguishing between implemented and prospective components underscores both the practical feasibility of the framework and its potential for incremental expansion and transferability to other epidemiological contexts.

## 6 Discussion

Combating the spread of any disease requires epidemiology, which reveals how and why it spreads, particularly when the disease is novel, as with COVID-19. As data on virus-related mortality and infections began to emerge, modeling of its spread was undertaken using several techniques. Government agencies used the spread modeling results to inform public health decision-making and policy updates, including rules on lockdowns, quarantines, use of face masks, and social distancing (23). Estimation of vital parameters to understand the range of possibilities of the virus has also been studied through research. There was initially limited data, but as more data were generated and made public, epidemiology revealed several possibilities, including viral transmission by people showing no symptoms (24), culminating in preparations for adverse conditions with forecasted exponential hospitalizations and admissions into intensive care. Epidemiological research conducted for previous infectious disease outbreaks (25) helped to inform such actions. Epidemiological research continues to illuminate the nature of the disease and the scale of the pandemic. As more variants emerge, epidemiology is helping public health professionals understand their potential effects and evaluate measures to contain the virus. The emergence of new variants has prompted new questions for epidemiologists, and we now have more data regarding their transmissibility and potential severity than earlier lineages of the virus (26). Hence, these new findings will require modifications to existing policy interventions made based on earlier data on transmission. Re-evaluation is necessary based on more recent data to determine which interventions to sustain and revise.

Furthermore, the development of vaccines for the virus has prompted epidemiologists to assess their impact on global spread. Epidemiology needs to establish the contribution of vaccines to increased immunity so that public health policymakers can determine whether to relax measures put in place to curtail the spread. Interestingly, the COVID-19 pandemic has altered epidemiology, such that several fields now have direct involvement in studies related to the pandemic and the virus. Epidemiologists collaborate across borders and time zones, sharing their data via various platforms to give scientists real-time and early access to results. The expansion of epidemiology has opened the door to contributions from researchers in other fields, including computer science, physics, and mathematics. Hence, it is necessary to find means of aggregating data, transforming it into a unified ontological model, defining automated links and relationships among data, and fostering data reasoning and the generation of insights. This is aimed at ensuring that researchers communicate their data and findings in a transparent manner and ensure the highest standards of research and data ethics. It will enhance the use of epidemiological data by the public, policymakers, and the media (26). The involvement of researchers from other fields has led to some challenges, specifically in terms of epidemic modelling and forecasting, which depend on statistical approaches towards making probabilistic predictions from real-time data. Incomplete data and inconsistent categorization sometimes make these initial predictions inaccurate. The semantic framework proposed in this study has the potential to help public health professionals filter uncertainties and inaccuracies by leveraging interoperability with respect to the semantics or meaning of specific datasets. Semantic interoperability facilitates the definition of the true meaning of content from healthcare data services and research. It facilitates stakeholders’ unambiguous access to and understanding of data, as consensus on meaning is required in the exchange of data across systems.

Beyond immediate outbreak surveillance and response, the integration of heterogeneous epidemiological, clinical, and genomic data carries significant organizational and health-system implications. Recent studies have demonstrated how combining clinical, functional, and organizational data can support the anticipatory management of downstream disease burden. For instance, integrated data approaches have proven vital in identifying patients at risk of prolonged post-COVID-19 symptoms, such as exertional dyspnoea, and in mapping effective rehabilitation trajectories. Similarly, these multi-source data models have been used to redesign hospital care pathways as systems transitioned into the post-emergency phase, ensuring that resource allocation remains evidence-driven.

These examples underscore that the value of richer, interoperable data infrastructures lies not solely in the technical aspect but also in the organizational. By enabling the reconciliation of heterogeneous models and the explicit representation of uncertainty, the proposed semantic FAIRness framework provides a foundation for transparent communication between data scientists, healthcare providers, and policymakers. In the UAE context, this capability facilitates the earlier identification of emerging care needs and the reconfiguration of clinical pathways. Ultimately, the framework supports informed decision-making under conditions of uncertainty, strengthening health-system resilience and ensuring that the UAE is better prepared to manage both the acute and long-term consequences of infectious disease threats.

While the current focus is often on genomic and clinical data, a truly multi-dimensional public health strategy must also integrate behavioral and attitudinal dimensions. Systematic reviews on COVID-19 and influenza vaccination hesitancy among diverse groups, including parents, healthcare workers, and pregnant women, have demonstrated that institutional trust, cultural norms, and social context are often the primary drivers of health outcomes, even when high-quality biomedical tools are accessible.

The proposed semantic FAIRness framework is uniquely positioned to address this by incorporating Social Determinants of Health (SDoH) and attitudinal data into its ontological structure. Because the framework uses extensible semantic triples, survey data and population-level behavioral indicators can be linked directly to traditional epidemiological endpoints, such as infection rates and disease severity. This enables a more nuanced analysis of *why* certain interventions succeed or fail in specific sub-populations. By enabling the joint analysis of biological and social determinants, the framework provides policymakers with the evidence needed to design context-sensitive strategies, such as targeted communication campaigns that address the root causes of hesitancy and improve the real-world impact of public health interventions in the UAE.

The proposed semantic FAIRness framework is presented as a conceptual and architectural contribution and has not yet been fully implemented or empirically evaluated as an end-to-end system. While elements of the framework build on prior work and established semantic and data integration practices, claims regarding improved decision-making, uncertainty handling, analytical performance, or policy support should be interpreted as prospective. These anticipated benefits are grounded in theoretical considerations and evidence from related domains, rather than direct empirical validation within the present study. Future studies will focus on technical implementation, deployment on real-world datasets, and systematic evaluation of the framework’s impact on epidemiological analysis and public health decision-making.

## 7 Conclusion

This research focuses on enhancing the epidemiological analysis of COVID-19 data, using the UAE as a case study, in response to the growing volume and heterogeneity of data generated during the pandemic. The proposed semantic FAIRness framework is intended to address challenges arising from fragmented data sources and analytical approaches by leveraging FAIR principles and semantic technologies to support more coherent data integration and interpretation.

At a conceptual level, the framework is designed to facilitate comparison across heterogeneous datasets and analytical models, thereby enabling more transparent representation of uncertainty and supporting the exploration of probabilistic forecasting scenarios. By promoting semantically interoperable data representations, the framework has the potential to reduce reliance on isolated technological silos and to provide a shared conceptual basis for integrating epidemiological data from diverse systems. Such capabilities may support clearer communication of uncertainty and analytical assumptions among researchers, public health practitioners, and policymakers.

More broadly, the framework is intended to complement existing epidemiological and genomic practices by providing an architectural foundation for cross-domain data integration and standards-aware data sharing. Future work will focus on the technical implementation and empirical evaluation of the framework, as well as on exploring its integration with complementary approaches such as deep learning and explainable artificial intelligence. These extensions are expected to enhance further the framework’s ability to support evidence-informed public health analysis and decision-making in the UAE and beyond.
